# Integrating the norm activation model and theory of planned behaviour to investigate farmer pro-environmental behavioural intention

**DOI:** 10.1038/s41598-023-32831-x

**Published:** 2023-04-05

**Authors:** Moslem Savari, Hamed Eskandari Damaneh, Hadi Eskandari Damaneh, Matthew Cotton

**Affiliations:** 1 Department of Agricultural Extension and Education, Agricultural Sciences and Natural Resources University of Khuzestan, Mollasani, Iran; 2 Department of Reclamation of Arid and Mountainous Regions, Faculty of Natural Resources, University of Tehran, Karaj, Iran; 3 School of Social Sciences, Humanities and Law, Teesside University, Middlesbrough, UK

**Keywords:** Ecology, Physiology, Ecology, Environmental sciences

## Abstract

Sustainable agriculture requires cooperative and coordinated action across multiple sectors and policy domains. However, farmer-stakeholder behaviors and action remain pivotal to sustainable food system management in many rural development contexts. We assess farmer pro-environmental behavioral intention through the development and application of a novel integrated approach combining two dominant psychological theories of behavior change: the Norm Activation Model (NAM) and Theory of Planned Behavior (TPB). We apply this framework to targeted research with potato growers of Kerman Province in southeastern Iran, using survey data (sample n = 381) analyzed through structural equation modeling (SEM). The integrated NAM-TPB model provides insight into both pro-social and self-interested motivations for farmer pro-environmental behavioral intention, with the model explaining 77% of total variance. We found that three variables, Awareness of Consequence (AC), Perceived Behavioral Control (PBC), and Subjective Norms (SN) were the strongest indicators of pro-environmental behavioral intention. We recommend therefore that agricultural extension and state-led farmer education should first emphasize awareness-raising of negative environmental impacts of current farming practices within training programs, and second, improve social learning amongst farmer communities through sustained farmer community engagement, thus “stabilizing” a social norm of environmental protection amongst peer networks of agricultural workers.

## Introduction

Agriculture is an essential part of every country's economy and plays a decisive role in income, employment, and food security globally^1^. Agricultural soils are critical for the efficient production of crops and safe food to meet the needs of a growing population^2^. However, improving soil quality is a critical component of sustainable agriculture^3,4^. Given the socio-economic and political pressure to improve soil fertility and increase agricultural productivity, widespread chemical fertilizer use, beginning in the 1950s and 1960s, led to increased food production at significant environmental cost^5,6^. Chemical fertilizers and pesticides have improved short-term food production^7–10^. However, soil degradation, greenhouse gas emission increases, and water pollution risks have emerged through their widespread use^11–16^. Consequently, excessive use of chemical fertilizers negatively impacts human health throughout the food chain^17^. For example, excessive use of phosphate fertilizers can lead to cadmium pollution, which if ingested can lead to osteoporosis^18^. Excessive use of nitrogen fertilizer leads to the accumulation of nitrites in plants; nitrites combine with amines increasing the risk of cancers of the digestive system, and methemoglobinemia in severe cases^17^. Fertilizer use leads to surface runoff and groundwater pollution^19,20^ contributing to eutrophication and consequently the deterioration of natural ecosystems and reduction of genetic diversity^21^.

Given growing global awareness of the adverse environmental impacts of agricultural intensification through pesticide and fertilizer use, alternative approaches such as agroecology, permaculture, naturalistic exploration, conservation agriculture, and low-input agriculture have become important agricultural policy and environmental management priorities^22^, in order to achieve simultaneous action on Sustainable Development Goal 2 (zero hunger) and Goals 14 and 15 (life on water and land respectively)^1^. Though the environmental, socio-economic and cultural challenges associated with sustainable agriculture require a holistic response from consumer organizations, food processing, export and retail organizations and agricultural governance institutions; in a development context one specific group of interest is the farmer-as-stakeholder within broader environmental and food systems. There is a growing literature on the relationship between farmer perceptions, intentions, pro-environmental values, behaviors, and practices^23–25^. Based on existing evidence, there is an apparent attitude-behaviour gap between declared environmental values and actual sustainable agricultural practices^26^, and thus environmental challenges are exacerbated by short-term profit-seeking at the expense of long-term common-pool resource management^27,28^. The psychology of human performance^29^ and examination of stakeholder behavioral patterns^30–32^ are thus key issues for sustainable development planning. The alleviation of environmental pressure through behavioral change is thus of growing interest to development scholars and practitioners^3,33,34^. Of particular interest in this study are the policy insights gained from understanding the environmental behaviors of rural agricultural stakeholders^35,36^. The novelty of this study lies in the integration of two behavioral models to explore behavioral intention amongst farmer-stakeholders—the Norm Activation Model (hereafter NAM) and the Theory of Planned b = Behavior (hereafter TPB)—to yield such agricultural and environmental policy-relevant insight.

## Theoretical framework and hypotheses development

### Norm Activation Model (NAM)

The Norm Activation Model (hereafter NAM), developed by Schwartz (1977), identifies the drivers influencing human intention towards altruistic and pro-environmental behaviors^37^. Pro-environmental behavior is often depicted as a type of pro-social behavior in the sense that it leaves positive effects on others^38^ and includes behaviors that reduce a person's destructive effects upon natural systems that are shared across multiple human and non-human communities^39^. Within the NAM model, behaviors/intentions are a function of personal norms (PN), which in turn, are regulated by awareness of consequence (AC) and ascription of responsibility (AR)^40^.

NAM posits that behavior begins with a person's awareness of the consequence of a destructive behavior, followed by developing a sense of responsibility regarding the adverse consequences of that behavior, and then ultimately raises the person’s intentions to act in a pro-social manner^41^. Behavioral intention is thought as a functional relationship between PN, AC, and AR^42^. AC activates the PN because when people become aware of their negative consequences upon others, it creates a sense of commitment^40^. AC makes a person aware of the positive effects of pro-social and pro-environmental behaviors on others^43,44^. AR involves a person's sense of responsibility for the consequences of pro-social and pro-environmental behaviors toward others^42^. PN reflects a sense of moral commitment to do, or not to do, certain actions that lead to pro-environmental behaviors^45^. PN is used as the most important variable of the NAM model to predict individual behaviors^42^. In general, while people are aware of the negative consequences of their behavior on others (i.e., AC) and hold themselves responsible (i.e., AR), they would be engaged with PN behaviors which in turn directly influence the individuals' intention^46^. NAM model have been applied in case studies of various pro-environmental behavioral contexts^47–49^, including those related to travelers^48^, convention attendees^47^ and tourists^48^.

**Figure 1:**
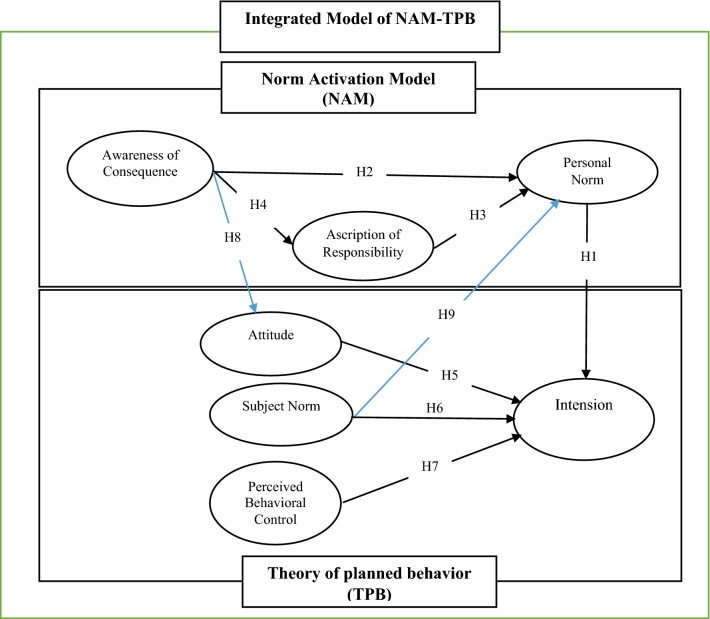
Shows the interaction between these hypothetical positions across the two models.

There is some debate regarding the relationship and order to the NAM variables, and how they affect one another^42^. The original NAM treats these variables as successive and linear (i.e. awareness of consequence → ascribed responsibility → personal norm → behavioral intentions)^38^ and this has been corroborated by empirical evidence^47,48^. However, a second viewpoint depicts that AC and AR can directly influence PN which initiates pro-environmental intentions and behaviors^38^. A third viewpoint hypothesizes that AC and AR modulate the relationships between PN and pro-environmental intention, indicating that the contribution of PN to pro-environmental intention is stronger in a group of people with higher levels of AC and AR^50^. Previous studies in this field have yielded contradictory findings due to confusion between different approaches^48^. However, recently it has become more common to use a mediating model, such that PN has a significant influence on performing pro-environmental behaviors and plays an intermediate role between AC and AR. Such a role can also be taken by AR between AC and PN^42,43,46,51^. We adopt a similar intermediate model in the present research (Fig. 1) hypothesizing the following:

- **H1**: The farmers’ PN toward pro-environmental behaviors has a significant influence on their intention.
- **H2**: The AC of pro-environmental behaviors has a significant influence on the farmers’ PN.
- **H3**: The farmers’ AR toward pro-environmental behaviors has a significant influence on their PN.
- **H4**: The AC of pro-environmental behaviors has a significant influence on farmers’ AR.

### Theory planned behavior (TPB)

The TPB model was proposed by Ajzen^52^ and has emerged as a highly popular socio-psychological theory for examining behavioral intentions across various research fields^50^. TPB is an extension of the Theory of Reasoned Action^53^ which, with the addition of Perceived Behavioral Control (PBC), has been applied to the prediction of a variety of human behavioral intentions^54^. The TPB model expresses the complexities between human behavior and its determinants, and importantly, implies that human behavior is the result of his/her intentions and tendencies^55^. TPB remains valuable for human behavioral intention study^56–59^ because the motivational factors identified in this model exert a strong effect on behavioral intention^52^. Intention is used here to describe an individual's beliefs regarding a particular behavior and is dependent on three factors: subjective norm(s) (SN), perceived behavioral control (PBC), and attitudes toward the behavior^44,60,61^. Attitudes refer to the degree to which an individual evaluates a specific behavior positively or negatively^62^. In general, an attitude is a set of beliefs regarding the consequences of a behavior^63^. In the TPB, attitude affects behavior, not directly, but through behavioral intention^64^. The SN refers to the perceived external social pressure to perform a behavior or not^46,65^. In other words, it refers to an individual’s perception of how much others approve or disapprove of their behavior^41^. Therefore, people's understanding of the affirmation of behavior by others within their social network can provide sufficient motivation for intentional behavior^66^. The relative strength of the SN therefore mediates the likelihood of a specific behavior^65^. The third component of the model, PBC, shows a person's belief in his/her ability to succeed in a behavior^42^, i.e. the perceived ease or difficulty of performing a behavior^67^. PBC therefore also mediates an individual’s intention to perform a particular behavior^33,43,44,50,68,69^.

TPB is a potent, widely applied theory of determining environmental behavioral intention^70–72^ and environmental variances^15,73,74^. In general, the variables of this theory, including attitude, SN and PBC, primarily govern the intention to engage in pro-environmental behaviors^75^. The hypotheses derived from TPB are as follow (Fig. 1):

- **H5**: Farmers' attitudes toward pro-environmental behaviors have a significant impact on their intention.
- **H6**: Farmers' SN toward pro-environmental behaviors have a significant impact on their intention.
- **H7**: Farmers' PBC toward pro-environmental behaviors have a significant impact on their intention.

### Merging NAM and TPB

NAM, rooted in pro-social behavioral intention and TPB based in self-interested motivation are widely adopted in the environmental psychology literature^64,76,77^, though there are specific advantages to applying the two models in tandem in order to improve responsiveness^47,64,65^, data depth^37,78,79^ and hence to better explore the farmers’ intention toward pro-environmental behaviors^47,80^. The principle advantage of a combined model is that it allows consideration of both pro-social and self-interested motivational factors in concert with one another^43,81^, and hence more rounded insights into pro-environmental behavioral context than their independent application^37,80,82,83^. By integrating NAM and TPB models we assess the dynamism among four variables of an AC, Attitude, SN, and PN.

Within the literatures combining these models there is a general consensus on the positive and significant association between Attitude and AC^41,43,46,84^. For example, Meng and Choi^84^ found that tourists' awareness of environmental consequences stimulated a positive attitude toward pro-environmental behavior. We posit therefore a similar hypothesis that farmers' awareness of the positive consequences of pro-environmental behaviors will result in favorable judgments about that behavior (noting that AC reflects a person's attitude toward a particular subject^46^. It has been also shown repeatedly that AC plays a leading role in the attitude of farmers toward pro-environmental behaviors^43,46,47,80^. The hypothesis of this section is therefore as follows:

- **H8**: Farmers' AC toward pro-environmental behaviors has a significant impact on their attitudes.

Second, the causal link between subjective and personal norms has been highlighted in the literature^37,80,82,83,85^. SN is “superior” to PN because SN expresses society's view of a behavior and determines whether the behavior is perceived as positive or negative. In other words, the broader social and behavioral norms of society will tend to guide an individual to recognize whether a behavior is appropriate or not^37,64,86,87^. There exist special standards in society that are exerted through social pressures and ingrained in individuals as PN^88^. Therefore, if farmers perceive that pro-environmental behaviors are socially acceptable, they may then feel a personal responsibility to perform those behaviors^43,89^. The next hypothesis was accordingly derived as follows:

- **H9**: Farmers' SN toward pro-environmental behaviors has a significant impact on their PN.

## Methodology

### Statistical population and sampling method

**Figure 2:**
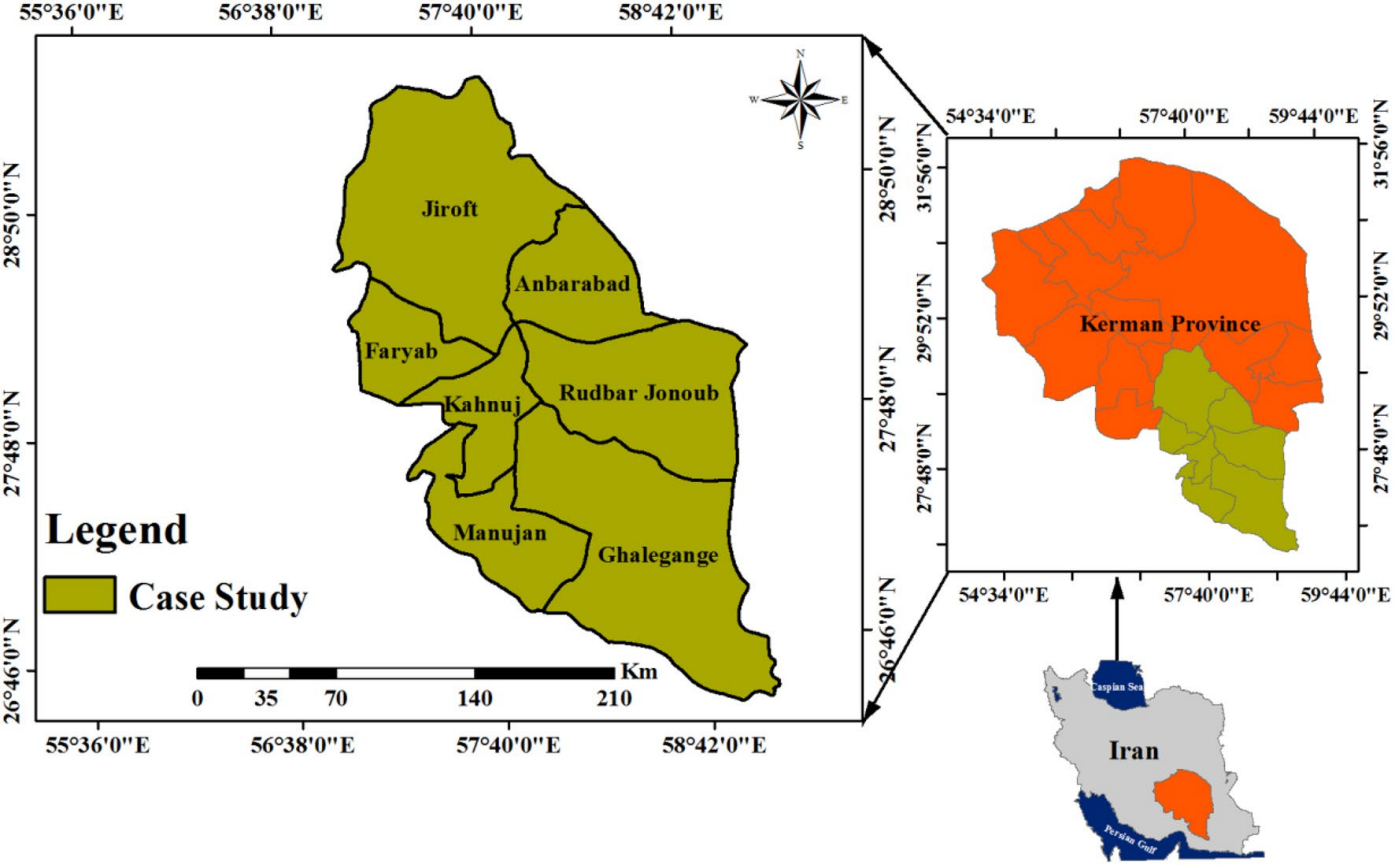
Study Area. ArcGIS software version 10.1 (https://www.esri.com/enus/arcgis/products/arcgis-pro/resources) was used to generate the figure.

The statistical population of the study included all potato growers of Kerman Province, (Jiroft, Anbarabad, Faryab, South Rudbar, Kahnooj, Ganj Castle and Manojan) in seven counties of the province (Fig. 2). Using Krejcie and Morgan table^90^, the number of samples was estimated at 381 individuals. We use a multi-stage stratified sampling method with the proportional assignment. Using this method, the sample percentage of each county was first determined based upon the proportion of the province’s total potato cultivation area. Based on the potato cultivation area, the percentage of each county sample was determined from the total sample, then from each county, four villages that had the highest cultivation area were selected for the study.

### Study area

The case study region is Kerman Province located in SE Iran. It has an estimated area of 111.285 km^2^, accounting for over 11% of total land, making Kerman the largest province of Iran. The province’s lakes are Jazmurian Lake, Bafgh Swamp, Gav-Khooni Swamp, and the watershed of the Lut Desert which are rapidly drying due to water resource mismanagement and climate change impacts.

Within this region, one of the most important agricultural products is potatoes, and this study specifically examines the perspectives of potato farmers. Iran is the 13th largest potato producer in the world, with a yield in 2018 produced across 5149 hectares of land. Potato varieties cultivated in these counties include Santa, Banba, Geely, Colomba, Silvana, Ernida and Bourne^91^. These varieties are produced in seven southern counties of Jiroft, Anbarabad, Faryab, South Rudbar, Kahnooj, Ganj Castle and Manojan amounting to 150 thousand tons of potato per year. Potatoes have become an increasingly important aspect of agricultural policy. Iran has traditionally been a heavy importer of rice, barley, and corn, though under conditions of US sanctions, global agribusiness have become reluctant to sell these products to Iran. As such, the Iranian government has increased domestic potato crop production to around 5 million metric tons since 2015, to provide a reliable source of energy-dense carbohydrates to Iranian citizens^92^.

Although these seven counties are rich in agricultural resources, producing more than 4.5 million tons of agricultural products per year, the excess use of energy, groundwaters, and fertilizers by their farmers has undermined the quality of soil and vegetation in these areas^93^. Moreover, poor-quality irrigation systems in these areas have wasted soil resources by increasing the soil’s harmful solutes and salts and led to desertification in this province, currently accounting for 35% of the total province’s area^94^. As such, a stronger understanding of famer perspectives on the relationship between an increased need for agricultural product volumes against ever-worsening environmental conditions due to agricultural practice is crucial if long-term sustainable food and environmental protection policy is to be successful.

### Measurements

We employed a research questionnaire which involved two elements. First was to ascertain the characteristics of individuals and their farms and, second, we proffered 25 items to determine the components of the integrated NAM-TPB model across seven sub-sections: (i) three items for measuring intension^20^ four items for PBC, (iii) four items for attitude, (iv) four items for SN, (v) three items for AC, (vi) four items for AR, and (vii) three items for PN. Respondents were asked to state the extent of their agreement or disagreement with statements made to measure the variable (based on Likert scale data, 1 [very low] to 5 [very high])^95^, adapting measurements used in an integrated NAM-TPB model from previous studies (Table 3).

#### Statement

All interviewees were informed about data protection issues by the enumerators and gave their consent orally at the beginning of each interview. Informed consent was obtained from all individual participants included in the study. All materials and methods are performed in accordance with the instructions and regulations and this research has been approved by a committee at Agricultural Sciences and Natural Resources University of Khuzestan, Mollasani, Iran. All procedures performed in studies involving human participants were in accordance with the ethical standards of the institutional research committee and with the 1964 Helsinki declaration and its later amendments or comparable ethical standards.

### Validity and reliability of instruments

In order to evaluate the indicators, the draft framework and questionnaire were piloted, reviewed and confirmed by an academic panel consisting of environmental science, psychology, social sciences and agricultural sciences expertise, before deployment of the questionnaire in the field. In addition, we used the Cronbach's alpha coefficient and composite reliability coefficient to evaluate the reliability of the research questions (Table 3).

### Data analysis

The field-collected data were analyzed by SPSS_23_ and Smart Pls. Our use of structural equation modeling (SEM) was due to its relative advantage in providing a robust multivariate analysis technique from the multivariate regression family. Generally, SEM is a combination of structural and measurement models^96^. More specifically, our approach is an extension of the General Linear Model that allows scientists to simultaneously test an array of regression equations. Another advantage is that it considers the measurement error into the analysis^67^. Smart Pls offers a quantitative and theoretical form of data analysis^97^.

### Informed consent

Informed consent was obtained from all individual participants included in the study.

## Results

### Descriptive results

The results of farmer and farm characteristics are presented in Table 1. The mean age of respondents was 48.82. The majority had an elementary level of education, accounting for 28.34% whereas those with a college/university degree were lower, at 13.91%. The average number of household members and years of agricultural experience were 3.85 individual and 31.35 years respectively. The average monthly household income was US$46.76, and the mean land area allocated for potato cultivation was 4.02 ha. Nearly 80% of respondents admitted to having never participated in any training or education related to pro-environmental behaviors or practices (Table 1).

**Table 1:** Demographic characteristics of farmers.

Variable	Category	Frequency	Percent	Mode
Age	Lower than 30	104	27.29	
	30–50	187	49.08	*
	More than 50	90	23.63	
Education	Illiterate	85	22.30	
	Elementary	108	28.34	*
	Secondary	72	18.89	
	High school	63	16.56	
	College education	53	13.91	
Number of household (person)	Lower than 3	108	28.34	
	3–4	174	45.66	*
	More than 4	99	26.00	
Monthly income (dollars)	Lower than 40	91	23.88	
	40–60	175	45.93	*
	More than 60	107	30.19	
Work experience	Lower than 20	74	19.42	
	20–40	205	53.80	*
	More than 40	102	26.78	
Under cultivation of wheat (Ha)	Lower than 3	102	26.77	
	3–5	155	40.68	*
	More than 5	124	32.55	
Presence in relevant training courses	Yes	85	22.30	
	No	296	77.70	*

### Descriptive statistics of observed variables

The results of the descriptive statistics on the condition of the observed variables among the studied farmers show that only two variables, Attitude and SN, were above the average (3 theoretical medians). All other variables were not (see Table 2).

**Table 2:** Descriptive statistics of observed variables.

Variables	Mean	Sd
Intention	2.66	0.711
Attitude	3.02	0.624
SN	3.11	0.648
PBC	2.74	0.633
AC	2.51	0.715
AR	2.35	0.627
PN	2.41	0.587

### Inferential statistics

In this section, the proposed conceptual model was presented in two sections: evaluation of the measurement model and evaluation of the structural model using the Partial Least Squares approach.

### Measurement model

The confirmatory factor analysis (CFA) of various indices were used to assess the validity and reliability of research components, including AC, AR, PN, Attitude, SN, PBC and Behavioral Intension, with values presented in Table 3 which indicate that the model had a good fit.

**Table 3:** Summary of goodness of fit indices for the measurement model.

Fit index	SRMR	D-G1	D-G2	NFI	RMS-theta
Suggested value	< 0.1	> 0.05	> 0.05	> 0.90	≤ 0.12
Estimated value	0.09	0.356	0.462	0.93	0.08

#### Uni-dimensionality

The results showed that the standardized operating load value (ƛ) of the selected indicators exceeded 0.669 and was significant at P < 0.01 which confirm their uni-dimensionality and acceptable accuracy for assessing the research components (Table 4).

**Table 4:** Results of confirmatory factor analysis for the measurement model.

Constructs	ƛ	t
Intention: ^46,52,103^: AVE = 0.795, CR = 0.904, Cronbach's alpha = 0.843		
I intend to adopt pro-environmental behaviors on my farm (Int1)	0.904	61.181
I like to adopt pro-environmental behaviors on my farm (Int2)	0.831	21.711
I am planning to adopt pro-environmental behaviors on my farm (Int1)	0.876	17.883
Attitude: ^98,99^: AVE = 0.791, CR = 0.938, Cronbach's alpha: 0.912		
It is wise to adopt environmental protection activities without harming the environment (Att1)	0.872	28.150
It is important to adopt environmental protection activities without harming the environment (Att2)	0.903	41.066
It is essential to adopt environmental protection activities without harming the environment (Att3)	0.923	41.995
It is helpful to adopt environmental protection activities without harming the environment (Att4)	0.859	31.085
Subject Norm: ^100–102^: AVE = 0.644, CR = 0.878, Cronbach's alpha: 0.817		
I have to adopt pro-environmental behaviors because friends, relatives and neighbors ask me to (SN1)	0.804	16.123
I have to adopt pro-environmental behaviors because other farmers believe I have to (SN2)	0.739	14.795
Society expects me, as a farmer, to adopt pro-environmental behaviors (SN3)	0.839	31.416
People who are important to me, such as agricultural experts, ask me to adopt pro-environmental behaviors (SN4)	0.825	17.889
Perceived Behavioral Control: ^101,103^ : AVE = 0.520, CR = 0.811, Cronbach's alpha: 0.815		
I believe I can adopt pro-environmental behaviors (PBC1)	0.804	13.810
I have the knowledge and skills to adopt pro-environmental behaviors (PBC1)	0.739	3.283
I believe I can adopt pro-environmental behaviors if I want to (PBC3)	0.839	3.468
I know how to adopt pro-environmental behaviors (PBC4)	0.825	3.670
Awareness of consequence: ^46,79^ AVE = 0.833, CR = 0.958, Cronbach's alpha: 0.933		
Performing pro-environmental behaviors reduces energy consumption and soil erosion (AC1)	0.946	79.000
Performing pro-environmental behaviors prevents loss of plants and animals (AC2)	0.953	86.850
Farmers' current practices could pollute the environment (AC3)	0.920	31.028
Ascription of responsibility: ^25,37,46^ AVE = 0.750, CR = 0.923, Cronbach's alpha: 0.888		
I think I am partly responsible for environmental degradation (AR1)	0.796	15.277
I think my farming methods are partly responsible for environmental degradation effects (AR2)	0.923	61.201
I think all farmers are responsible for protecting the environment (AR3)	0.882	34.988
I think I need to do something to protect the environment (AR4)	0.857	24.777
Personal Norm: ^43,46,104^ AVE = 0.567, CR = 0.795, Cronbach's alpha: 0.701		
I feel obligated to adopt sustainable farming methods (PN1)	0.868	24.941
If I don't destroy the environment, I will feel like a better farmer (PN2)	0.706	8.778
I feel I have a moral obligation to adopt pro-environmental behaviors (PN3)	0.669	7.512

#### Reliability and validity

The results showed that the composite reliability and Cronbach's alpha coefficient values of the model's components were higher than 0.6 and 0.7, respectively. Also, the average extracted variance of all components of the proposed research model was more than 0.50. These results indicate that all latent variables of the proposed research model had good reliability and validity (Table 4).

#### Diagnostic validity

As shown in Table 5, the AVE of the research components (0.75 < AVE < 0.94) was greater than the correlation coefficients between them (0.31 < r < 0.54) which confirms the diagnostic validity of the components of the proposed model.

**Table 5:** Correlations with Square Roots of the AVE.

Constructs	1	2	3	4	5	6	7
1-Intention	0.87^a^						
2-Attitude	0.38**	0.92^a^					
3-SN	0.41**	0.37**	0.82^a^				
4-PBC	0.56**	0.33**	0.41**	0.93^a^			
5-AC	0.47**	0.52**	0.47**	0.47**	0.88^a^		
6-AR	0.54**	0.41**	0.53**	0.46**	0.31**	0.75^a^	
7-PN	0.47**	0.51**	0.47**	0.47**	0.44**	0.54**	0.94^a^

**Correlation is significant at the < 0.01 level.

^a^The square roots of AVE estimate.

**Figure 3:**
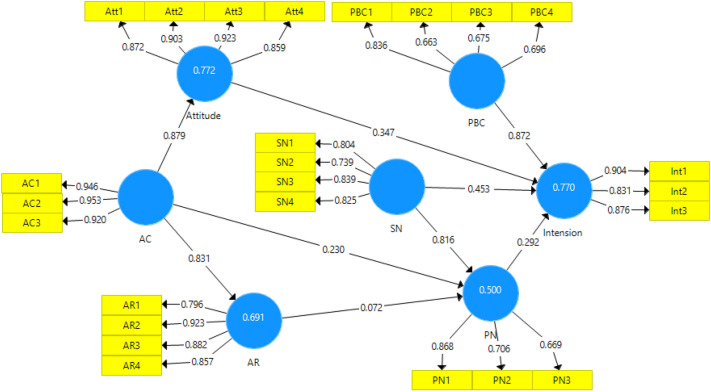
Path model with standardized factor loadings.

**Figure 4:**
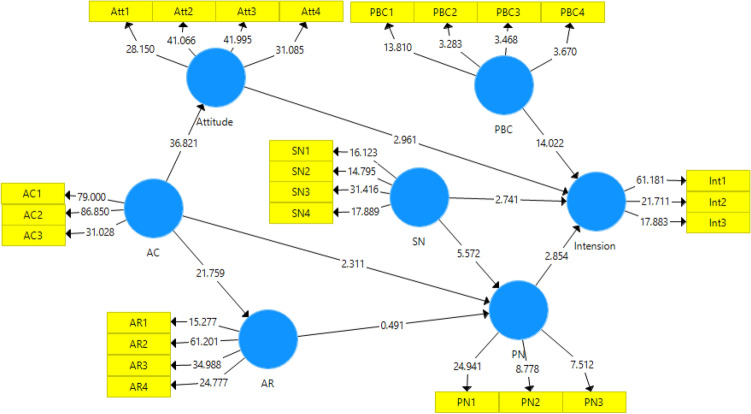
Path model with *t*-values.

After confirming the measurement models using CFA, the path analysis method (structural model evaluation) was used to test the hypotheses in the context of the proposed conceptual model. The research path model is presented by showing the standardized and significant factor loads in Figs. 3 and 4.

#### Research hypothesis test

The farm-level results of the final effect of the variables on farmers' intention to perform environmental behavior are presented in Table 6. The bootstrapping method was used to test the research hypotheses. The results showed that all research hypotheses were confirmed except for the effect of AR on PN. Moreover, research variables were able to explain 77% of farmers’ intention to perform pro-environmental behaviors.

**Table 6:** Results of research structural models.

Hypothesis	ƛ	t	Result	R^2^
H1: PN ➞ intention	0.292	2.854	Confirm	0.77
H2: AC ➞ PN	0.230	2.311	Confirm	
H3: AR ➞ PN	0.072	0.491	Reject	
H4: AC ➞ AR	0.831	21.759	Confirm	
H5: attitude ➞ intention	0.347	2.961	Confirm	
H6: SN ➞ intention	0.453	2.741	Confirm	
H7: PBC ➞ intention	0.872	14.022	Confirm	
H8: AC ➞ attitude	0.879	36.821	Confirm	
H9: SN ➞ PN	0.816	5.572	Confirm	

## Discussion

In this study, two psycho-social models were integrated to investigate farmers' tendencies to perform pro-environmental behaviors. In line with the findings of^41,42,46,48^, the results of SEM showed that PN significantly influenced the farmers' behavioral intention (Confirm Hypothesis 1). These studies found that PN is the most important and strongest predictor of behavioral intention in the NAM model because PN refers to the relative perceived rightness and wrongness of behavior in individuals^44,65,105^. Hence, if farmers recognize pro-environmental behaviors as right, they would be more likely to perform these behaviors^41^. For example, if farmers feel that the use of chemical fertilizers on their farm is harmful, and then perceive that the behavior is by extension harmful to the community, then they avoid this behavior because of PN-related disapproval^106^. In this study, positive emotions indicate the farmer's sense of satisfaction with performing environmental protection behaviors while negative emotions induce a sense of guilt due to not performing pro-environmental behaviors.

The results of the effect of AC on PN supported hypothesis 2 and were consistent with the findings of^37,77,80,83^. According to these results, it can be concluded that awareness of the consequences of pro-environmental behaviors can affect people's normative commitment. In fact, AC can activate PN in performing or not performing a behavior ^40^. Therefore, gaining awareness of the environmental impact of pro-environmental behaviors is the first step toward achieving the principle of sustainability, and is essentially a prerequisite for the future survival of humanity^42^, because AC brings environmental problems to the forefront of personal decision-making, whilst its neglect would limit pro-environmental behavioral intention^27^.

The results of hypothesis indicate that, contrary to research^37,46,80,83^, AR does not have a significant effect on PN. In the interpretation of this finding, we assert that farmers renting land will prioritize short term agricultural profitability at the expense of the negative environmental consequences of their actions. Profitability is sought above the stabilization of pro-social, pro-environmental community norms. AR cannot create specific PNs because economic factors, especially in developing countries like Iran, take precedence over other factors, and personal benefit over collective benefit due to high poverty and low economic power often prevail^107^. The second important interpretation of hypothesis 3, is shown in the descriptive statistics—more than 50% of the farmers have a high level of education. Education level is associated with pro-environmental behavioral intention in that it both illustrates the level of awareness of environmental impacts, and the subjective social norms that regulate pro-social and pro-environmental behavior. It is therefore important for sustainable agricultural policy-making to ensure that awareness raising of environmental impacts is combined with social engagement within and between farmers in order to establish social norms of environmental protection and thus long-term practical environmental management change within food systems (see for example^46^.

The next confirmed hypothesis was the effect of attitudes on farmers' behavioral intention to perform pro-environmental behaviors Hypothesis, a finding in line with the results of^33,41,43,46,59,103^. It could be interpreted that attitude is usually known as a precondition for human behavior and is a key factor in revealing hidden behaviors in humans^33^. Therefore, having a positive attitude towards the environment determines behavioral tendencies in humans and is the key to farmers' behavior^41^. Given that attitude is the strongest variable in predicting behavioral tendencies^52^ and expressed valence judgments (negative and positive) about the consequences of a behavior^98^, the higher the value of people's judgments about pro-environmental behaviors, the greater the tendency to engage in those behaviors.

The results of this study corroborated those of^33,43,65,66^ which confirm the significant effect of SN on farmers’ behavioral intention (Confirm hypothesis 6). Accordingly, social pressures contribute significantly to forming behavioral tendencies in individuals^80^ (Park and Ha, 2014), because SN provides people with the information they need about how to apply right or wrong and useful or undesirable behaviors^44,80^. Also, if farmers feel that their behavior is approved by the community’s members with the highest social status, they are more likely to engage in that behavior. In other words, villagers who are encouraged by *authorities* and those with the greatest social capital, are more likely to believe in the morality and responsibility of pro-environmental behaviors^66,85^. The results of the effects of PBC on farmers’ behavioral intention was supportive of Hypothesis (7) and consistent with those of^33,42,43,59,68^. The higher a person's self-esteem and the belief that they are capable of pro-environmental action, the more likely they are to engage, because PBC illustrates the ease or difficulty of understanding a person's behavior^108,109^. In other words, pro-environmental behaviors are more frequent in farmers who believe that they possess the knowledge and skills needed to perform such behaviors^50^. In this respect, training courses such as locally run workshops might prove useful in capacity building and thus stimulating pro-environmental behavior change.

The result of the AC effect on farmers’ attitude and AR toward pro-environmental behavior supported Hypothesis 8, 4 and corroborated the findings of^41,43,46,84^. This finding indicates that knowing the consequences a behavior determines individuals’ attitude toward performing or not performing a behavior because attitude is dependent upon the positive and negative evaluation of a person about that behavior^84^. As such, farmers' awareness of the consequences of pro-environmental behaviors influences their attitude toward environmental protection^41^. For example, research on the use of media to show the effects of chemical fertilizer utilization on human health have been shown to reduce farmer chemical fertilizer usage through a process of attitudinal change^106^.

The result of the effects of SN on PN supported Hypothesis 9 and the findings of^37,65,80,82,83,110^. It depicts that social space plays a significant role in institutionalizing a behavior among members of society^86^ because when diffuse social pressures amongst a community emphasize the importance of specific behaviors, it becomes difficult for individuals to work against this dominant set of social norms^64^. Therefore, pro-environmental behaviors would likely occur among farmers even without direct supervision if more generalized social norm pressures govern these behaviors^88^. It is recommended therefore to work alongside those with the greatest social and cultural capital resources within rural communities to promote the uptake of pro-environmental behavioral norms.

## Conclusions

This study investigates the factors affecting farmers' tendency toward pro-environmental behaviors in Iran. We designed an integrative approach combining two psychosocial theories (TPB and NAM). The integrated NAM-TPB modeling reaches higher performance than either of the two applied singularly because the combined procedure accounts for both socially-motivated and self-motivated drivers of behavioral change in a way that studies using an individual model do not^37,44,70,79^. Our results show that the integrated NAM-TPB model can explain 77% of farmers’ intention towards adopting pro-environmental behavior; indicating the significant impact of this socio-psychological model in explaining farmers' intention towards sustainable agricultural practices.

We find that the key variable influencing farmer adoption of pro-environmental behavior is awareness of consequence, and that excessive agricultural environmental exploitation is driven by a paucity of farmer-stakeholder knowledge around environmental impacts, and hence a lack of capacity to plan sustainably. The concept of capacity-building through knowledge exchange concerning the relationship between personal action, agricultural policy strategy under conditions of economic stress and long-term environmental change, are thus a key priority for Iranian governance bodies. Such insight is applicable to a range of agricultural development contexts across the world. In Iran, as elsewhere in the developing world, agricultural policies prioritize short-term economic and food quantity benefits, such that actions including increasing fossil-fuel based fertilizer and pesticide inputs, and the burning of plant residues are promoted through policy ‘supply push’ by central government authorities. These policy actions are aimed at improving annual yields and ensuring food security under conditions of external pressure (in the case of Iran under conditions of economic sanction), alongside declining productivity under conditions of water stress, heat stress and extreme weather events such as floods resulting from anthropogenic climate change. Yet as is commonly understood by agricultural and environmental scientists, agricultural intensification without remediation action degrades the quality of the common pool land, water, and ecosystem service resources over time. That farmers are largely unaware of this fact is a key concern for sustainable food-systems management. We recommend in this case, that educational and training activities such as workshops and extension courses to articulate these longer-term impacts in the context of farm livelihood sustainability would provide a key means to ensure uptake of pro-environmental behaviors and agricultural practices. However, in Iran, as in other developing nations, the public sector takes responsibility for agricultural skill development. If state policies remain focused on short-term productivity and self-sufficiency, then this creates a stable social norm of “short-termist” agricultural development. The *moral* responsibility for long-term sustainable development of the agricultural sector therefore lies with central government in terms of both their agricultural supply and education policies. Improvements to the ethically motivated and pro-social commitment of farmers to sustainable agricultural practices can only be made under conditions in which supply chain management and farmer training both emphasize pro-environmental behaviors and practices in concert with one another.

If a combined supply chain and farm education strategy that emphasizes long-term sustainable agricultural planning can be implemented then this research shows that there would then be little need for direct monitoring of farmer activities, as social pressure will institutionalize sustainable practices as persistent social norms amongst farmer stakeholder networks and rural communities. The advantage of such an approach is that it produces considerably less social and economic cost to government, as there is less need for formal regulatory oversight and coercive control to manage environmental impacts. Such savings could instead be used to provide low-interest loans or subsidy to farmers to purchase green fertilizers and modern irrigation technologies, or to diversify rural economies away from intensive agricultural development to stimulate sustainable practices in line with emergent pro-environmental social norms.

## Data Availability

Due to data protection and participant confidentiality concerns, datasets generated during and/or analyzed during the current study are available from the corresponding author upon request.
